# CETS: Enabling Sustainable IoT with Cooperative Energy Transfer Schedule towards 6G Era

**DOI:** 10.3390/s22176584

**Published:** 2022-08-31

**Authors:** Raja Sravan Kumar Kovvali, Gopikrishnan Sundaram

**Affiliations:** School of Computer Science and Engineering, VIT-AP University, Amaravati 522237, India

**Keywords:** wireless energy transmission, broadcasting, radio frequency energy harvesting, wireless powered internet of everything, cooperative scheduling

## Abstract

The large scale of the Internet of Things necessitates using long-lasting physical layer devices for data collection. Deploying large numbers of Wi-Fi-enabled devices is expensive, so the Internet of Everything (IoE) is equipped with multiple communication modules to collect data where Wi-Fi is unavailable. However, because of their extended communication capabilities, IoE devices face energy limitations. As a result, IoE devices must be provided with the necessary energy resources. This paper introduces a novel multi-hop cooperation communication mechanism for Wireless Energy Transfer (WET) in the Wireless Powered-Internet of Everything (WP-IoE). IoE devices are outfitted here with various communication devices such as RF, Bluetooth, and Wi-Fi. This research proposes a two-phase energy transmission schedule to address the energy requirements. For data collection, the first phase provides a distributed tree-based data communication plan. The proposed model’s second phase used the reverse data collection protocol to implement wireless energy transmission. By combining these two phases, an optimized WET framework was created without unmanned aerial vehicles or robots. The experimental findings show that the proposed method in this research increases the average lifetime of the network and has a more significant charge latency and average charge throughput than other models.

## 1. Introduction

The term “Internet of Things (IoT)” refers to connecting all physical places and objects across the world to the Internet. When something is connected to the Internet, it can send, receive, or sometimes transmit and receive information. When objects can transfer or receive data, they become intelligent. The Internet of Things (IoT) revolution has a lot of clever promises for bettering people’s lives. It is seen as a critical technology enabler in developing smart cities, communities, and households. Every second, 127 product innovations connect to the Internet, and Cisco projects that by 2030, there will be 500 billion connected gadgets [1]. In addition, IoT could offer advanced connectivity of heterogeneous components to form a single system in the context of energy systems [2]. However, there are significant difficulties in smoothly integrating multiple domains.

The Internet of Things has an increasing demand for long device service lifetimes. In practice, continuously supplying enough power to IoT devices is a critical concern. The IoT device lifespan can be extended by upgrading or replacing them. However, the cost of charging batteries may be prohibitive or inconvenient. In other cases, such as when you are unable to contact someone, it is impossible to connect those devices once they have been installed [3]. However, the problem with these battery-operated wireless sensor systems is that it would be physically impossible to replace the batteries of billions of devices spread over the globe, let alone space. Not only that, but the short lifespan of batteries would cause network operations to be disrupted [4]. Currently, the issue regarding energy in the Internet of Things (IoT) industry, IoT sensors with low energy consumption and long battery life are required, i.e., a sensor’s battery must last 10 to 15 years [5].

**Figure 1:**
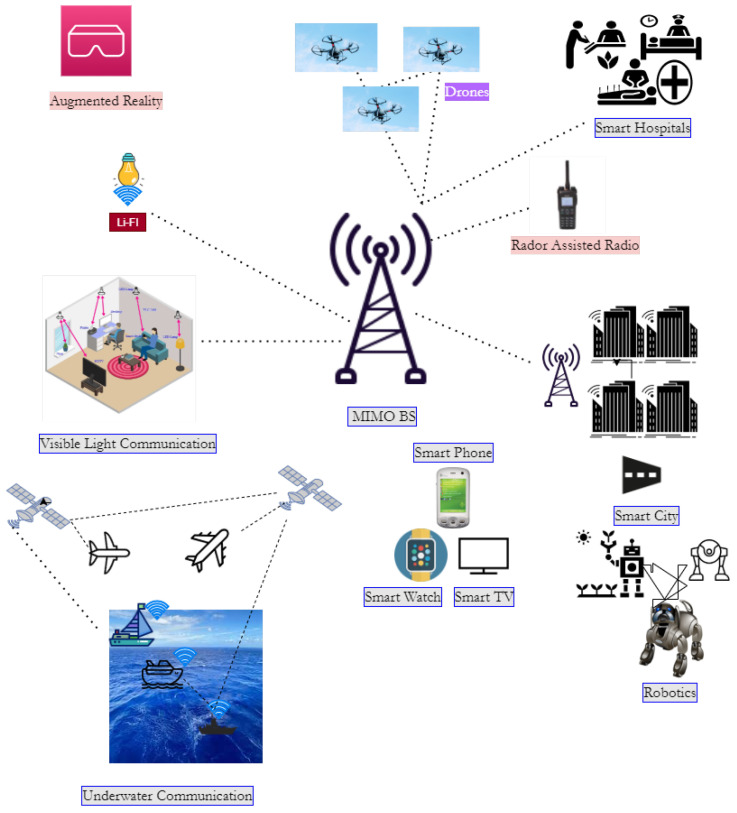
Communication paradigm for IoE devices.

Since 1980, approximately every ten years, a new wireless mobile communications generation has emerged, with the 1st generation analogue FM cellular schemes in 1981, the 2nd generation in 1992, the 3rd generation (3G) in 2001, and the 4th generation (4G) (often known to as the long-term evolution (LTE)) in 2011 [6]. The 5G is growing its importance, and the framework is being deployed globally [7]. Now, after 5G, A lot of work is being done to characterize and understand wireless networks beyond 5G, which call the sixth generation (6G) of systems [8] as shown in Figure 1.

Nowadays, the Internet of Everything (IoE) is the subject of great discussion because of the rapid development of IoE-based smart applications. The Internet of Everything (IoE) refers to the use of new technologies to connect things, data, people, and processes to provide a wide range of smart services. Autonomous linked vehicles, brain-computer interfaces, extended reality (XR), vehicles, and haptics are examples of emerging IoE services. The majority of these services rely on ultra high reliability, large data rates, unmanned mobility management, and long-distance communication [9], meet the requirements of this new class of services, specific challenges should be addressed, such as characterizing the fundamental rate-reliability-latency tradeoffs that govern their effectiveness. Wireless devices must become self-sustaining, intelligent network fabric that adaptively provisions and orchestrate communication computing control—localization—sensing resources. To address these issues and accelerate the deployment of new IoE services, a disruptive sixth-generation (6G) wireless system is being developed, with an architecture that is normally tuned to the performance standards of the aforementioned IoE applications and telecommunications networks [10]. In this paper, the research addresses the cooperative energy transfer schedule in the Internet of Everything.

Following that, the paper is structured as follows: Section 2 provides the literature review, Section 3 discusses the proposed model CETS, Section 4 discusses the results, and Section 5 provides conclusions.

## 2. Literature Review

As 5G technology matures and becomes more commercialized, more researchers are turning their attention to 6G [11]. The 6G technology will feature a greater transmission frequency, and a higher maximum data rate than 5G [12]. Lower latency, improved dependability, and new applications, such as AI-assisted intelligent communication and vehicle network intelligence, are all possible with 6G [13]. A large number of IoT devices (IoTDs) must be installed in the 6G wireless communication network to support these unique 6G applications [14].

A 6G system will enable a variety of smart applications by utilizing novel communication technologies. Terahertz communication, quantum communication, 3D wireless communication, visible light communication, nanoscale communication, and holographic communication are a few instances of these communication technologies [9]. In terms of energy efficiency, the ultimate objective is a green society aided by 6G networks, particularly load balancing [15], zero-energy/cost/emission Internet-of-Things (IoT) deployments. In addition, the scientific community and industry see energy harvesting (EH) approaches as a viable option for externally charging batteries or avoiding battery replacement [3]. However, because of several other factors, this remains a big concern due to a scarcity of mature solutions for powering and maintaining the huge number of gadgets that will continue to operate without interruption [16]. Ref. [17] proposed the hybrid tree-based criteria for aggregation of data in wireless networks.

Wireless Energy Transfer (WET) has a lot of potential for replacing or extending the life of batteries. RF-EH devices can become self-sustaining in terms of the energy required for operation, allowing them to operate indefinitely while requiring minimal maintenance [18]. Ref. [19] studied on Simultaneous Wireless Information and Power Transfer technology for WET in 6G. Ref. [20] focused on Energy transfer strategies using RF-enabled sensors to transmit energy from one node to another in a network. In [21], the authors proposed two chargers approach for energy transfer through wireless networks. The authors of [22] created a Radiofrequency MAC protocol to optimize energy transmission to wireless sensors while lowering data transmission disruption. RF-MAC is based on the (CSMA/CA) protocol and includes the following processes: assessing the charging threshold in maximum, selecting relevant energy transponders for getting charged, seeking and granting energy transfer user requests, and finding the respective data transmission and power transfer priorities. In references [23,24], the authors focused on the NOMA methodology for transferring energy in wireless systems.

In reference [25], the authors propose an energy-efficient method for NOMA grouping selection. They show a user clustering technique in a NOMA-based framework for incorporating a cooperative scheme while also maximizing communication efficiency based on the use of WET. Some works have focused on optimizing the transmitted power to reduce energy consumption. In reference [26], the authors jointly optimize the time coursework for offloading and Energy Harvesting, as well as the transmission energy at the gadget for offloading. Similar works can be found in reference [27]. In reference [28], a game-theoretical approach to the resource routing scheme in a wireless network for IoT applications is proposed. They attempt to achieve the best possible energy transfer both for access points (AP) as well as the harvesting devices. Authors [29,30] proposed on MISO SWIPT and SWIPT on intelligent reflecting surfaces in WET. Ref. [31] worked WET on multiple devices, whereas [32] worked on WET on distributed antennas. In [21], the author compares NaZCCS (a naive version of zoning and collaborative charging scheduling) to ZCCS (zoning and collaborative charging scheduling). Only global chargers are taken into account for energy transfer in NaZCCS, whereas in ZCCS, both local and global chargers are taken into account. Ref. [33] focus on the impact of network partitions and build a new “Multi-chargers region Partition charging Scheduling Algorithm (MPSA)” related to network information. The authors investigated the impacts of various partitions and partition methods on whole sensor networks and compared different MCs scheduling algorithms from multiple perspectives such as the usage of effective energy efficiency and the sensor nodes’ survival rate in the whole network. Ref. [34] suggests a “Distributed Mobile Charging Protocol (DMCP)” for scheduling numerous mobile chargers (MCs) on large-scale wireless rechargeable sensor networks. Here Sensor nodes are partially rechargeable under the DMCP based on their requests to MCs. The authors turned the MCP into repeated games played by the MCs. They expressed each game as a 0–1 ILP model for the minimal delay in charging and more extensive charging coverage. Refs. [35,36] proposed load balancing in terms of energy in heterogeneous IoT networks.

### 2.1. Motivation

In the current scenario, WET is performed by UAVs (Unmanned Aerial Vehicles) such as drones [37] or other robots or toy types of vehicles. As a result, they will move throughout the network and transfer energy from one node to all existing nodes, necessitating the use of additional hardware for energy transfer. The problem statement demonstrates how our model can transfer energy without the use of unmanned areal vehicles.

### 2.2. Problem Statement

Many research solutions for energy efficiency have been proposed to handle the energy limitations of wireless sensor devices. However, these solutions limit the capabilities of these devices. Alternatively, many energy harvesting solutions have been proposed and successfully implemented. This harvesting solution broadens the range of WSN applications to include WSN-enabled IoT, Radio Frequency (RF), and Energy Harvesting (EH) via Wireless Energy Transfer (WET). This WET propels WSNs into a new network paradigm: wireless-powered communication networks (WPCN). This WPCN drew many contributions from researchers and evolved into the Wireless Powered Internet of Everything (WP-IoE). However, the successful implementation of this WP-IoE is fraught with deployment challenges. This study proposes a new multi-hop prediction-based cooperation communication scheme for WET in WP-IoE.

In this model, the research considers a continuous Energy supply station known as a Base Station (BS), from which the energy is transmitted to a network made up of nodes (N), some of which are connected with Radio Frequency (RF), others with Bluetooth (B), suntil others with Wireless Fidelity (W), and suntil others with IoE nodes to transfer energy from one node to another in the network. The energy was transferred from the source node alias BS (which will have a good energy resource and continuous power supply) to all other devices via the predicted shortest path. The novelty of our work is that instead of deploying additional hardware, the model is going to develop a communication protocol between one node and another node so that a node with a higher energy capability will act as a redistribution node to transfer energy from one node to another and named it a redistribution node because it has more energy and can quickly transfer energy to other nodes in the network that require energy to stay alive. The issue is that, because there will be many nodes in the network, determining to which node the energy needs to transfer is a critical condition that must be met. To address this issue, this research used a technique known as broadcasting. Therefore, in our proposed model, a tree-based approach for transferring energy from one sensor node to another has been proposed so that the approach shows the optimal way to make all the network nodes alive in the network.

### 2.3. Contributions

- This research proposed a distributed tree-based WET schedule algorithm for transferring energy from one node to another with no additional hardware.
- A broadcasting algorithm has been proposed for transferring energy, which transfers energy to the needy node to keep the node alive in the network.
- The proposed model includes the construction of an Internet of Everything (IoE) network capable of transferring energy not only to IoT devices but also to non-IoT nodes in the network.

## 3. CETS: Cooperative Energy Transfer Schedule

### 3.1. Distributed Tree Based WET Schedule Algorithm (DTS)

**Figure 2:**
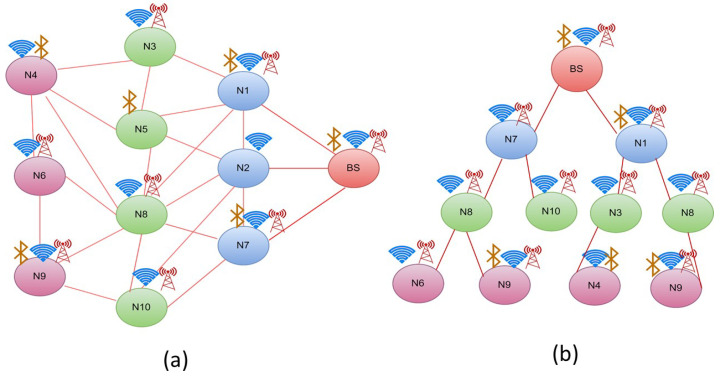
(**a**) Sample Network and (**b**) DTS.

The tree construction method used by DTS is a two-hop tree construction method. Initially all hops should perform broadcasting so that the node which performs broadcasting will discover its neighbouring nodes along with their available energy and communication modules. Following the neighbour discovery phase, the data tree construction begins at each node. The sink node (Base Station (BS)) with constant energy is the root node in charge of gathering data from the sensor nodes. As a result, the base station first recognizes the neighbour node with the fewest communication modules and assigns that node to the left. It then locates the node with the second smallest number of communication modules among all other reachable nodes and adds it as the right child. The proposed tree approach is a binary search tree and a node in a binary search tree can only have two children. As a result, the constructed control can now be shifted to the left child of the BS. The left child node now employs the same binary search tree technique as its right and left child nodes, as shown in Figure 2.

This process will be repeated until all of the nodes are reached. Construction regulation will advance to the next node or level only after all of the node’s left and right child nodes have been completed or if a node has only one left child. The construction phase will halt once a node has discovered all of its member nodes and their parent nodes.

Algorithm 1 is used to divide the entire network into a binary tree so that the congestion of the network will be reduced, and thereby the energy transfer can be done efficiently. The tree has been constructed using this algorithm based on the communication modules. Fewer communication modules will be placed on the left, and more communication modules will be placed on the right to form a binary tree. The sample network shown in Figure 2a is converted as the tree as shown in Figure 2b. The tree construction details and the nodes’ positions are shown in the Table 1, Table 2, Table 3 and Table 4.

**Algorithm 1** Distributed Tree Based WET Schedule Algorithm

**Require:**
List of Neighbour NodesCurrent nodeDistance


**Ensure:**
Broadcasting of all Nodes

$Status_{Node}<-Not-visited$



$Node_{dist}<-0$



Current−Node<−B1



**Begin**
1:Neighbour-Node List of Current-Node($N_{1}$,$N_{1dist}$,$N_{1ener}$), ($N_{2}$,$N_{2dist}$,$N_{2ener}$), ⋯, ($N_{n}$,$N_{ndist}$, ⋯, $N_{nener}$)2:Current-node-distance = 13:**if** List-count-Neighbour-Node ≥ 1 **then**4:    $N_{L}\leftarrow Minimal-Distance-Node\left(Neighbour-Node-ListB_{1}\right)$5:    $N_{L}$-Status = Visited6:    $N_{L}$-Distance = Distance-17:    $N_{L}$-Root = Left8:
**end if**
9:**if** List-count-Neighbour-Node ≥ 2 **then**10:    $N_{R}\leftarrow Second-Minimal-Distance-Node\left(Neighbour-Node-ListB_{1}\right)$11:    $N_{R}$-Status = Visited12:    $N_{R}$-Distance = Distance-113:    $N_{R}$-Root = Right14:
**end if**
15:**while** Distance is greater than zero **do**16:    Current−Node<−MinimalDistance(MD)17:    Update Neighbour-Node-List of Currnet-Node18:    Current-Node-Distance = 119:    temp = MD20:    **while** temp ≠ 0 **do**21:        **if** First-Node in Neighbour-Node-List = Visited **then**22:           Pop First Neighbour from the List23:           MD = MD -124:        **else**25:           MD = MD-126:        **end if**27:    **end while**28:
**end while**





### 3.2. Energy Model

The designed multi-hop energy transfer network, as shown in Figure 2, is made up of a network as a graph (G) with Nodes (N) and Vertices (V). The model is structured so that the first node is designated as the Base Station (BS), with BS in V, and the other nodes are designated as Sub Nodes (SN) or Intermediate Nodes. This BS will have a constant energy supply in comparison to other nodes. It also outperforms other nodes in the network in terms of computational speed. Table 5 shows the system parameters used to construct equations. The nodes that require energy to be alive are called End Nodes, the SubNodes that supply energy to the End Nodes are referred to as Intermediate Nodes, and the Base Station is referred to as Access Point.

Given a time *t* instance, the total consumed energy at the end node at that time *t* is given by Equation (1)

(1) $$E_{com}^{EN}\left(tot\right)=E_{T}^{EN}T_{t}^{EN}+P_{R}^{EN}T_{r}^{EN}+P_{com}^{EN}T_{c}^{EN}+P_{i}^{EN}T_{i}^{EN}$$

such that *t* = $T_{t}^{EN}$ + $T_{c}^{EN}$ + $T_{i}^{EN}$ + $T_{r}^{EN}$.

The harvested energy of the end node is calculated using Equation (2)

(2) $$E_{T}^{EN}=D_{com}*E_{T}^{RN}*T_{tx}*E_{tx}$$

where $D_{com}$ represents all the Communication Modules in the network. $T_{tx}$ is the time taken to transmit data. $E_{tx}$ is the Consumed Energy of a node.

All communication modules (Bluetooth nodes, Radio Frequency Nodes, Wireless Fidelity Nodes, and Wireless Development Module) are used in this research. The amount of energy consumed will vary depending on the communication module installed at a node. As a result, the energy consumption of a node is calculated as shown in the equations from (3) to (6).

(3) $$E_{tx}\left(BT\right)=\frac{OP_{E}\left(BT\right)*Data}{D_{com\text{-}Throughput}}$$

(4) $$E_{tx}\left(RF\right)=\frac{OP_{E}\left(RF\right)*Data}{D_{com\text{-}Throughput}}$$

(5) $$E_{tx}\left(WiFi\right)=\frac{OP_{E}\left(WiFi\right)*Data}{D_{com\text{-}Throughput}}$$

Equation (3) is used to compute the energy consumption ($E_{tx}\left(BT\right)$) of a Bluetooth enabled sensor node, where $OP_{E}\left(BT\right)$ is the operational energy of a Bluetooth-enabled node.

The data are the size of the data required to send from one Bluetooth-enabled sensor node to another.

$D_{com\text{-}Throughput}$ is the amount of data successfully transferred from one Bluetooth-enabled sensor node to another in a given time (*t*).

The Equation (4) is used to calculate the energy consumption (Etx(RF)) of an RF-enabled sensor node, where OPE(RF) is the operational energy of an RF-enabled node.

The data size required to be sent from one RF-enabled sensor node to another is called data.

$D_{com\text{-}Throughput}$ is the amount of data successfully transferred from one RF-enabled sensor node to another in a given time (*t*).

The Equation (5) is used to compute the energy consumption ($E_{tx}\left(WiFi\right)$) of a WiFi enabled sensor node, where $OP_{E}\left(WiFi\right)$ is operational energy of an Internet of Things (IoT) node.

The data are the size of the data required to send from one WiFi-enabled sensor node to another.

$D_{com\text{-}Throughput}$ is the amount of data successfully transferred from one WiFi-enabled sensor node to another in a given time (*t*).

The Total Energy harvested over a time (*t*) is given by the Equation (6)

(6) $$H_{E}^{EN}\left(t\right)=H_{p}^{EN}\left(t-T_{t}^{EN}-T_{r}^{EN}\right)$$

Here, the transmission time $T_{t}^{EN}$ depends on available bandwidth and the size of the data [38], which is given by Equation (7)

(7) $$T_{t}^{EN}=\frac{D_{com\text{-}Throughput}}{r}$$

where $D_{com\text{-}Throughput}$ is the amount of data successfully transferred over a link, and *r* is the data rate.

The received energy $\left(E_{R}^{EN}\right)$ is the same as the transmission energy $\left(E_{T}^{EN}\right)$ because whatever the recipient node will receive, the node harvests.

After calculating the consumed and harvested energy and assuming there is initial energy at the node, the residual energy at any given time is shown in Equation (8)

(8) $$R_{E}^{EN}=R_{E}^{EN}\left(0\right)-E_{com}^{EN}\left(tot\right)+H_{E}^{EN}\left(t\right)$$

where $R_{E}^{EN}\left(0\right)$ is the node’s initial energy.

Since the relay node or intermediate node communicates with the end node and the AP, its energy consumption differs from that of the end node. Simultaneously, a portion of its energy is expended in broadcasting this same energy to the end node via the wireless link. The total amount of energy consumed at the receiver node up to time *t* is given by (9)

(9) $$\begin{array}{cc} C_{E}^{RN}\left(t\right)= & \;E_{T}^{RN}\left(T_{t}^{RN-N}+T_{t}^{RN-AP}\right)+E_{R}^{RN}(T_{r}^{RN-N}+\\ & T_{r}^{RN-AP})+P_{com}^{RN}T_{c}^{RN}+P_{I}^{RN}T_{i}^{RN}+\\ & E_{T}^{EN}H_{t}^{EN} \end{array}$$

where

$$t\leq \left(T_{t}^{RN-N}+T_{t}^{RN-AP}\right)+\left(T_{r}^{RN-N}+T_{i}^{RN}+H_{t}^{EN}T_{r}^{RN-AP}\right)+T_{c}^{RN}$$

Here $T_{t}^{RN}$ and $T_{r}^{RN}$ are the transmission and reception time between the intermediate nodes and the end nodes. $T_{t}^{RN-AP}$ and $T_{r}^{RN-AP}$ are the transmission and the reception time between intermediate and the end nodes.

The Harvested energy at the Intermediate node or relay node is given by (10)

(10) $$E_{T}^{RN}=D_{com}*E_{T}^{AP}*T_{tx}*E_{tx}$$

and the remaining energy of the intermediate node is given by Equation (11)

(11) $$R_{E}^{RN}=R_{E}^{RN}\left(0\right)-E_{com}^{RN}\left(tot\right)+H_{E}^{RN}\left(t\right)$$

where $R_{E}^{RN}\left(0\right)$ is the node’s initial energy.

The Threshold value of the end node is given in Equation (12)

(12) $$Th_{E}^{EN}\geq E_{com}^{EN}\left(tot\right)$$

and the Threshold value of the Intermediate Node or relay node is given in Equation (13)

(13) $$Th_{E}^{RN}\geq E_{com}^{RN}\left(tot\right)$$

Here, the Threshold values will vary based on the communication modules.

### 3.3. Overview of Energy Transfer Schedule

- STEP 1: Find the Residual Energies of each node along with their communication modules.
- STEP 2: The BS is a continuous energy supply station node capable of all modes of communication, i.e., a WDM Node (WiFi, RF, and Bluetooth). Therefore, that it will transmit the energy based on all possible ways of communication, and the receivers will receive the energy based on their communication sensor embedded in their node.
- STEP 3: Here in the network, the leaf nodes will always have more energy when compared with intermediate hops.
- STEP 4: Whenever BS broadcasts the energy, it will request the receiver hops to broadcast their available energy along with their distance, once it gets the responses from the reachable hops, it will maintain a list of their residual energies.
- STEP 5: The communication modules decide the threshold. If a node has more communication modules, its energy consumption is more, and if it has few communication modules, its energy consumption will be less.
- STEP 6: The requesting node can get the energy either from the BS or from the neighbouring hops based on the node coverage region.
- STEP 7: If the requesting node gets the energy from the BS as explained in CASE 1, then there is no need to check for the sender’s threshold since BS is a continuous energy supply station, If the requesting node is receiving energy from the neighbouring hops as illustrated in CASE 2 then based on the neighbouring hops residual energy is concerned which hop is having more energy that node will be involved in energy transfer to the requesting node.
- STEP 8: The higher energy neighbouring node will transfer the energy until it reaches its threshold limit, once it reaches that limit, it stops transmission.
- STEP 9: If more than one neighbouring hop has the same energy and suppose both the hops have maximum energy, then their communication modules will be considered for energy transfer which was illustrated in CASE 3. Since both nodes will broadcast their energy simultaneously, and the receiver may have a chance of collision. The maximum communication modules hop will be considered for energy transfer to overcome this collision.
- STEP 10: If the receiver receives energy from the maximum communication modules hop, it will consider the sender hop and receives energy from that hop, and after that, if it receives energy from any other hop, it will discard it.

CASE 1: Energy requesting from Base Station (BS)

**Figure 3:**
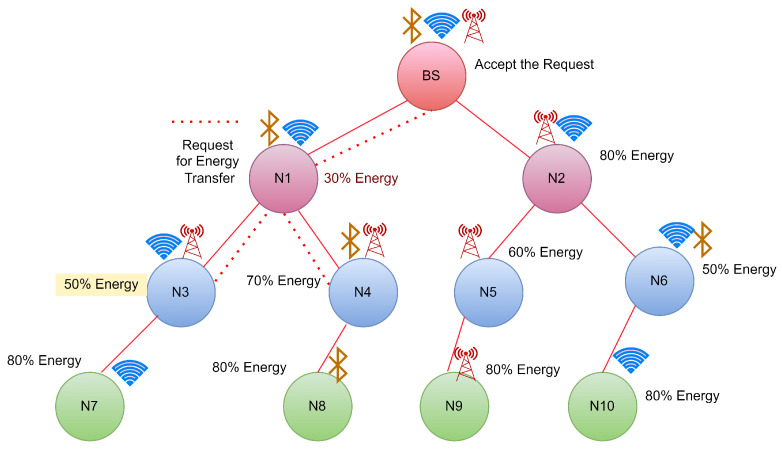
Case 1: Energy request from N1 to its neighbour hops.

**Figure 4:**
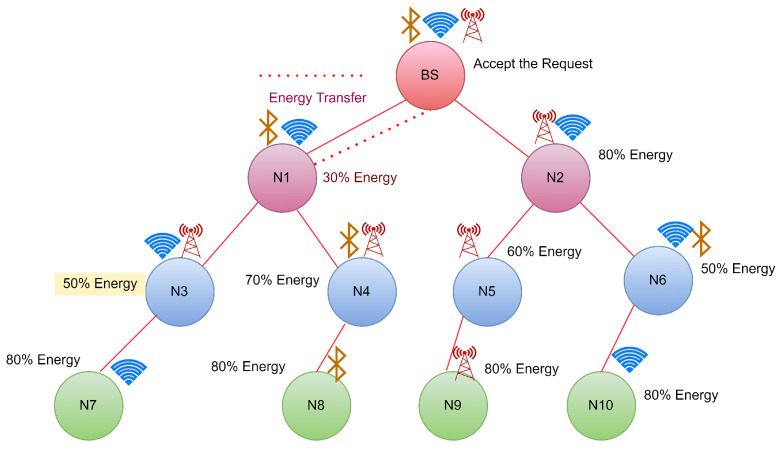
Case 1:Energy transfer from BS to N1.

In this case, Node N1 reaches the threshold shown in Figure 3, so it will send the broadcast request to its neighbour nodes for energy transfer. In this case, the BS will receive the request sent by N1. As BS is a continuous energy supply station, it will accept the request sent by N1 and start transferring energy to N1 until it gets fully charged, as shown in Figure 4.

CASE 2: Energy transfer from the neighbour hops

**Figure 5:**
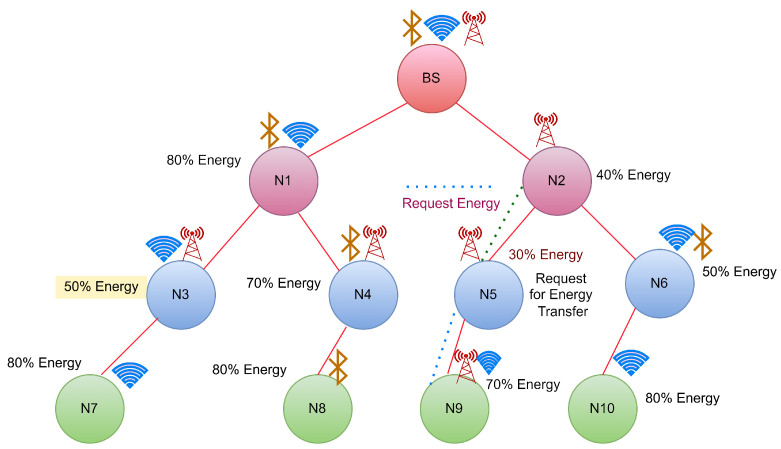
Case 2:Energy request from N5 to its neighbour hops.

**Figure 6:**
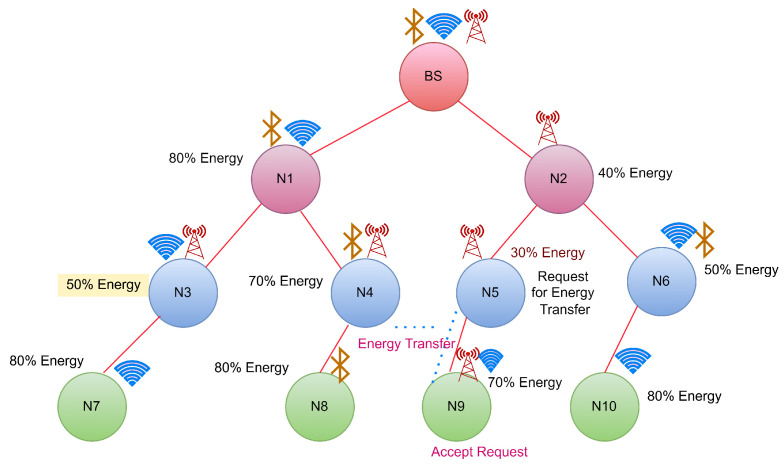
Case 2:Energy transfer from N9 to N5.

The node energies and their communication media are shown in Figure 5. As per STEP 5, if a hop reaches a threshold value, it should broadcast the request. In this figure, N3 is the hop which reaches the threshold value. Hence it will request the energy by broadcasting. Because N5 is not a neighbour of BS, it requests the neighbours to share energy based on their available energy. The neighbours of N5 are N2 and N9. The residual energy of N2 and N9 are obtained as 40% for N2 and 70% for N9. As N9 has more energy than N2, it will transfer the energy to N5, as shown in Figure 6 and Table 6.

CASE 3: Energy transfer to a node has equal energy neighbour hops.

**Figure 7:**
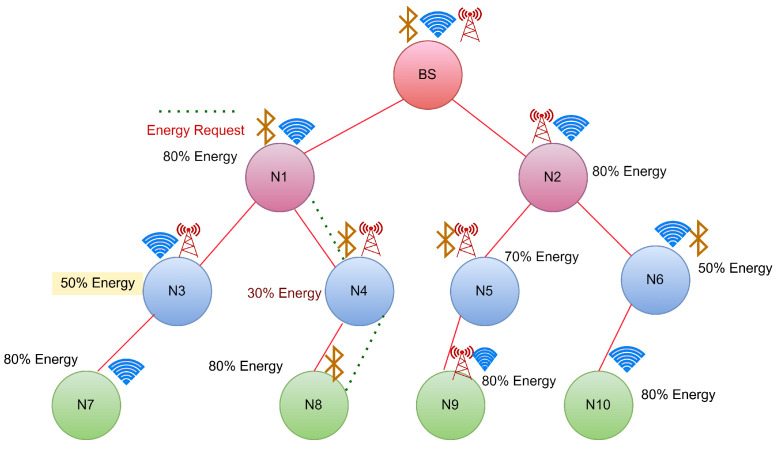
Case 3:Energy request from N4 to its neighbour hops.

In this scenario, if the requesting node is not a neighbour of BS and reaches a threshold, it will initiate an energy transfer request to its neighbour nodes as shown in Figure 7. If we suppose the neighbouring nodes have the same energy, then both of them will start transferring the energy to the requested node, and this might cause a collision so that the requested node may not receive energy from any of the nodes.

**Figure 8:**
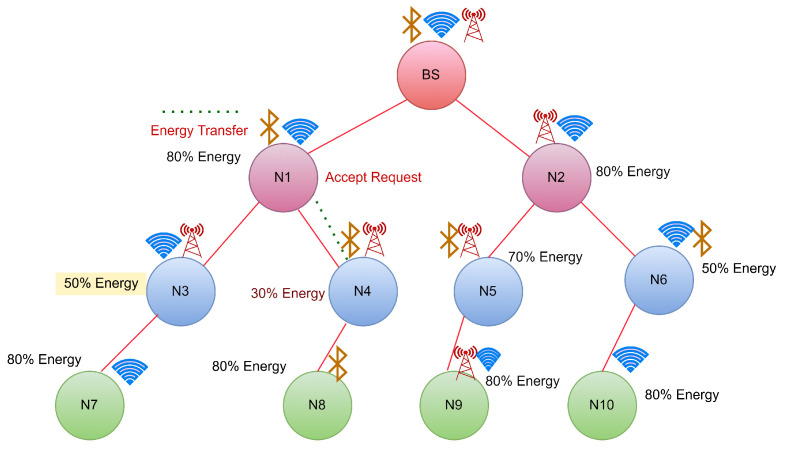
Case 3:Energy transfer from N1 to N4.

Node N5 has the least energy, while its neighbours N2 and N9 both have residual energy of 80%. Both of them will now transfer, which may result in a collision. To overcome this, the node from which the energy will be received first will be chosen for energy transfer, as shown in Figure 8.

### 3.4. Overview of CETS

The proposed Algorithm 2 Cooperative Energy Transfer Schedule (CETS) takes the input from the hybrid tree. After the tree is constructed, each node’s residual energy will be calculated using Equation (8) and will store in a list. The energy discovery of every node is shown in the energy discovery algorithm. After obtaining residual energies of all nodes, the minimum energy node will initiate an energy request for energy transfer to neighbouring nodes by broadcasting. In that process, the request sent by the node whose residual energy is less will be received by its neighbouring hops. Now the job will be on the neighbouring hops to decide who needs to initiate the energy transfer to the needy node. For that, the Algorithm Energy request to the BS explains that if the BS has received the request, the BS will transfer the energy to the needy node until the node energy becomes full. In another case, if the BS is not the neighbouring node of the needy node, then Algorithm Energy Request to the neighbour nodes will illustrate how the node residual energies are calculated. This algorithm will decide which node needs to proceed for the energy transfer and to what extent the node can transfer the energy. Algorithm conformation of node recharging will describe how the energy will be conformed for recharging the needy node.
**Algorithm 2** CETS**Input:** Hybrid Tree with root as BS from Algorithm 1**Output:** CETS.
1:**for** node(j) in N **do**//N is the number of nodes in the network.2:    list l = call Algorithm 33:    **for** i in l **do**4:        **if** l[i].RE < Threshold **then**5:           Broadcast Energy request6:           list l1 = neighbour nodes7:        **else**8:           Mark the node as energy transfer node9:        **end if**10:    **end for**11:    **if** BS is in l1 **then**12:        call Algorithm 413:    **else**14:        N = call Algorithm 515:    **end if**16:    Send request from node(j) to N17:    node(j).RE = call Algorithm 618:    **if** node(j).RE < Threshold **then**19:        repeat from step 1.20:    **end if**21:**end for**

$E_{tx}$ is the transmission energy which is the sum of transmission energy of Bluetooth, RF, and WiFi-enabled sensor nodes i.e., $E_{tx}=E_{tx}\left(BT\right)+E_{tx}\left(RF\right)+E_{tx}\left(WiFi\right)$ which is shown in equations from (3) to (5).

In Algorithm 3 each node in the network maintains a record of its available energy to initiate WET. Various functions, such as sensing, transmitting, and aggregation, determine the remaining energy of each sensor node. As a result of these functionalities, the energy utilisation rates at various sensor nodes vary. When the remaining energy of such sensors falls below a certain threshold, a charging request is sent to the SN. Because the sensor nodes are separated from the SN, charging requests can sometimes arrive at the SN in different orders, yet if they are carried out concurrently. An SN always fulfils a request in the residual energy series, which is decentralized and runs on each sensor node. The energy consumption of member nodes is proportional to their path length from the sender or recipient. Lines 2–4 of the algorithm monitor each sensor node’s remaining energy and when it falls below a certain threshold.
**Algorithm 3** Energy discovery algorithm**Input:** Wireless Energy Transfer Network(N)**Output:** Nodes Available Energy(node(j).RE)
1:**for** node(j) in N **do**2:    **if** node(j).RE > Threshold **then**3:        $E_{con}$ = $E_{tx}$+ data + distance4:        Node(j).RE = Node(j).RE − $E_{con}$5:    **end if**6:**end for**


Where $E_{con}$ is the consumed Energy of the node.

The Algorithm 4 is for a request for recharging if a node has a minimum energy problem, i.e., node(J).RE is less than the predefined threshold and sends a charging request to the BS. The node number and current energy status are included in this billing request. The charging request is recorded by the BS, which obtains the node’s ID and energy. The total number of registered charge requests is further utilized to evaluate the throughput charging. The BS schedules the charging of accepted requests according to node(j).RE, where j is the ID of the sensor node. Algorithm 4 depicts the recharge request mechanism for the nodes in the network.
**Algorithm 4** Energy request to BS**Input:** Energy request from node(j) to BS**Output:** Energy transfer to node(j) from BS
1:Request receive from node(j)2:Locate the node(j) position3:**while** node(j).RE ≠ FULL **do**4:    call Algorithm 65:**end while**6:Return


Similarly, when an SN’s energy is denoted as node(j). When the RE falls below a pre-defined threshold, it will forward a request for recharge to the neighbour nodes in the same process mentioned above. This charge request includes the subnode number and the subnode’s current energy. When the neighbour nodes receive multiple recharging requests, it organizes the recharging of neighbour nodes in ascending order of node (j). RE, where j is the ID of the subnode. If the neighbour node is preoccupied charging another node, the requesting node(j) begins the request for recharging procedure with its neighbours. Algorithm 5 illustrates this.

In Algorithm 6, node(j) requests for energy transfer to either BS or NN. If the BS is a neighbour to node(j), then until the node is fully charged, the BS will transfer the energy to node(j). In another case, if BS is not a neighbour to node(j), it will request its NN to transfer the energy. In Algorithm 6 based on the communication modules of the neighbouring nodes, the threshold value will be decided by the node and based on the threshold concerned, and the node will transfer to the node(j).
**Algorithm 5** Energy request to Neighbour-Nodes(NN)**Input:** Energy request from node(j)**Output:** Returns maximum residual energy node to node(j)
1:node(j) broadcast energy2:list l = NN[Id]3:**for** i in l **do**4:    list l1 = call Algorithm 35:**end for**6:**for** x in l1 **do**7:    **if** x > Max **then**//First node in the l1 as Maximum8:        Max = x9:    **end if**10:**end for**11:return Max


**Algorithm 6** Confirmation of Node Recharging
**Input:** node(j) request to Max or BS**Output:** Energy Transfer to node(j)
1:node(j) request to Max of BS2:**if** NN is BS **then**3:    **while** node(j).RE ≠ FULL **do**4:        Send the 1Eu of energy to node(j)5:    **end while**6:**end if**7:**if** Max == high or low communication module node **then**8:    calculate Threshold9:    **if** Max > Threshold **then**10:        transfer 1Eu to node(j)11:    **end if**12:**end if**



## 4. Results

### 4.1. Network Model

**Figure 9:**
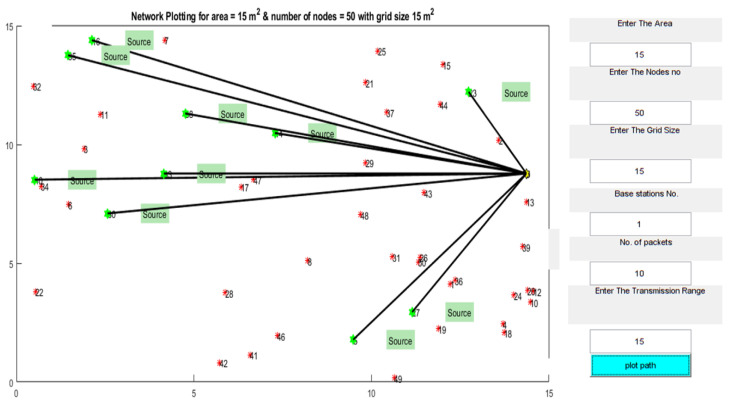
Network model.

In this research, the proposed methodology uses dual-energy chargers in the network. Base Station (BS) and Subnodes (SN) or hops. BS is stationed in the network in such a way that it will ensure that the energy is transmitted to all SNs in the network’s transmission range. The SNs are used to gather and accumulate sensor data before forwarding it to the other SNs in the network, which may consume additional energy. When compared to the sensor nodes, the leaf nodes have significantly more energy. Our investigations divide the simulation model area into four 15 × 15 m${}^{2}$ zones. The nodes in each region are assigned randomly, as shown in Figure 9. In this model the Yellow color node is the Base Station. The source nodes shown in green color are the charging nodes which were changing each time depends on the communication modules, and the other nodes are shown in red color. It is built to be self-sufficient in a network full of mobile nodes, withstanding phenomena like node movement, link failures, and packet drops. CETS keeps track of connections between nodes in the form of a routing table. There are three crucial pieces of information contained in the entry for a given destination in a routing table: the next hop node, the sequence number, and the hop count. Next-hop nodes are used to forward all data packets onwards to their final destination. A route’s "freshness" can be quantified by its sequence number, which functions like a time stamp. Distance to the final node is shown by the number of hops taken so far.

Each SN has a 15-meter charging range to cover its territory, with the premise that each SN can charge sensors in its immediate vicinity. If an SN’s energy consumption falls below a certain threshold, the SN requests on-demand recharging from the BS using Algorithm 4. Because each SN is supposed to be deployed at the region’s center, the communication range for BS is set to 15 to ensure that all SNs are recharged. This research considers the base station as the continuous energy supply station in the network model. It has all the communication media, i.e., it can communicate through WiFi or Bluetooth or RF; hence it has been called a WDN node. The other nodes in the network are either enabled with WiFi nodes, RF nodes, or Bluetooth nodes. The ranges and the transmission, receiving, data rate and Operating voltages of the nodes are shown in the Table 7. When the sensor nodes’ (SNs’) energy falls below a predefined threshold, they initiate a charge request. The charge request includes an ID of Node and its energy availability. Incoming charging requests are buffered and served in order of their remaining energy by SN.

### 4.2. Performance Evaluations

This section assesses CETS’s performance and compares it to the ZCCS, NaZCCS, MPSA, and DMCP. The reason for considering them are as follows. (1). For recharging the SNs, these methods use multiple MCs. (2). NaZCCS follows a partial charging model, whereas ZCCS, MPSA, and DMCP follow a full charging model. (3). DMCP is a game theory-based model. This may be observed that no current on-demand charging system uses a fully distributed model. Section 4.2 describes the performance measures used for the evaluations, and Section 4.3 provides performance evaluations for the experiments under various scenarios.

### 4.3. Performance Measures

The concept of charging throughput is introduced in this study to evaluate the effectiveness of CETS and compare it to the ZCCS, NaZCCS, MPSA, and DMCP. The hop count charged per unit time is referred to as charging throughput. To ensure optimum network longevity, CETS strives to maximize charging throughput. It ensures that only a limited number of nodes consume energy and are thus recharged quickly. In another way, charging throughput represents the average operating time of sensor nodes indirectly. Because of the dynamic environment of sensor networks, it is not easy to predict a sensor’s operating time; thus, performance evaluations consider evaluating the count of sensor hops charged per unit time. Compared to existing recharging schedulers, ZCCS, NaZCCS, MPSA, and DMCP operate on-demand and necessitate the exchange of various control packets between the sensor and SN and BS. Therefore, the transfer of these energy chargers may consume additional energy.

In this research’s tests, energy overhead is defined as the number of transmissions transmitted between sensors and charging hops to fulfil a 1 Eu charge request. An SN, such as a WET, must deliver 100 J pulses toward that sensor hop by magnetic resonant coupling to charge an energy unit (1 Eu = 50 J). The results are an average of five runs. The research also finds the average charging delay experienced by sensor nodes to determine how quickly CETS can recharge the energy of sensor nodes. The charging delay of a sensor node is defined as the time between when the hop submits the recharge request and when the sub-node starts charging.

### 4.4. Throughput Analysis

The charge throughput of CETS, ZCCS, NaZCCS, MPSA, and DMCP is compared in this section at various node and round densities. The number of sensor hops deployed per unit area is called node density. The node density is calculated in the experimental tests under consideration using the MATLAB function poissrnd. For example, an average of five rounds in an area of 60 × 60 with 45, 80, and 114 hops produce node densities of 0.1, 0.2, and 0.3. All outcomes are determined by the number of hops generated by the poissrnd function during a run.

**Figure 10:**
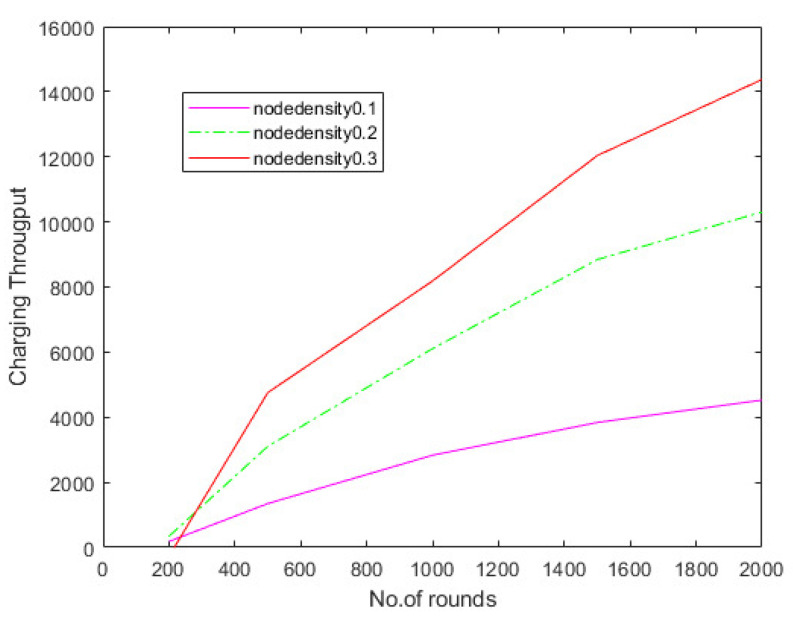
Comparative analysis of charging throughput for CETS at various node densities.

**Figure 11:**
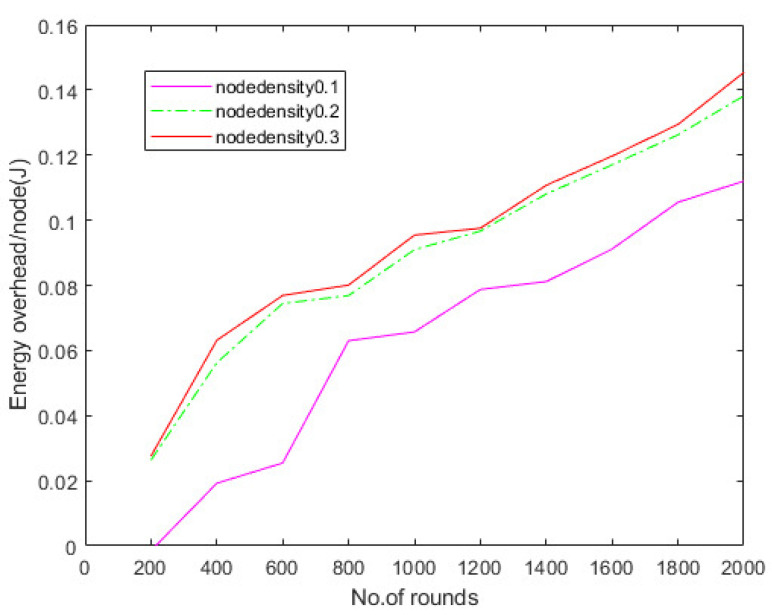
Comparative analysis of energy overhead for CETS at various node densities.

**Figure 12:**
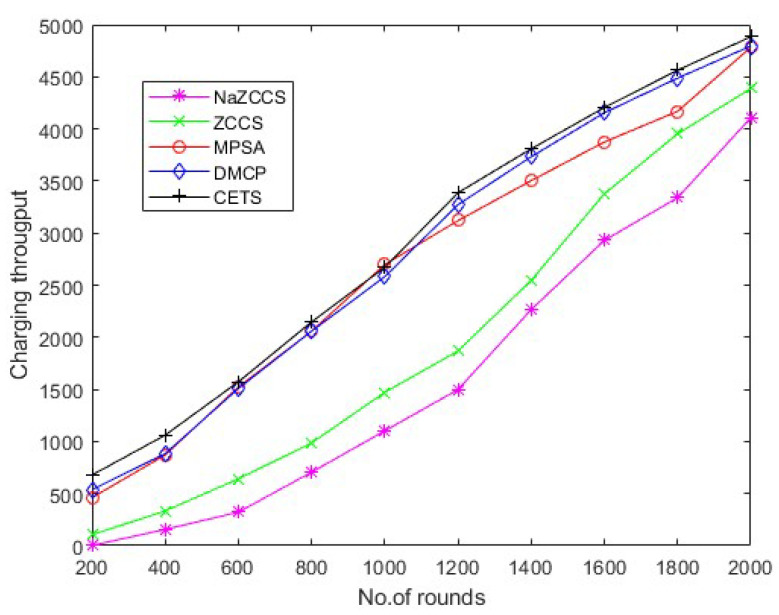
CETS versus other models on charging throughput per round at hop density 0.1.

**Figure 13:**
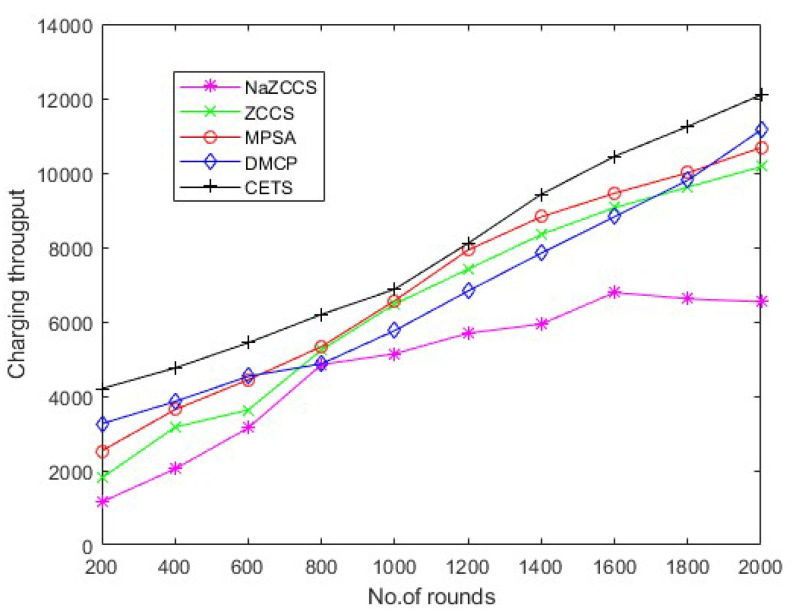
CETS versus other models on charging throughput per round at hop density 0.2.

**Figure 14:**
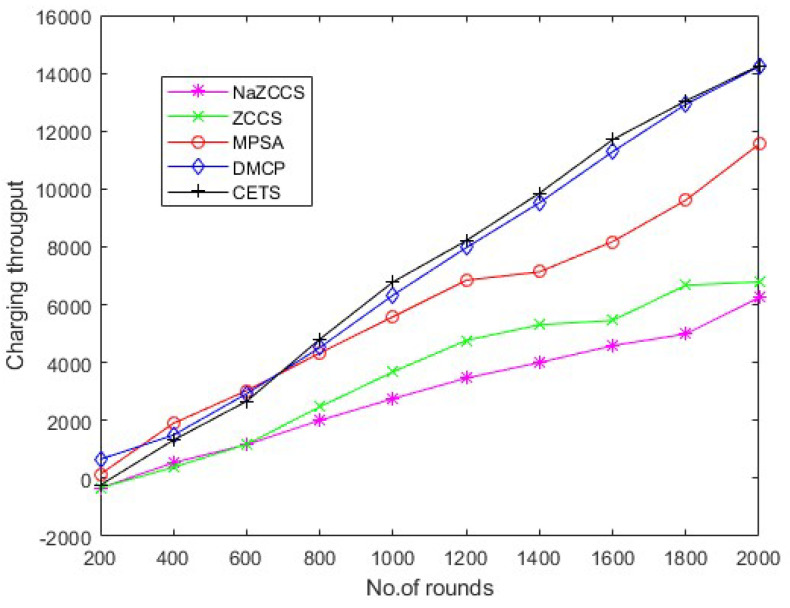
CETS versus other models on charging throughput per round at hop density 0.3.

Hierarchical communication via wireless sensor networks is possible, involving multiple rounds of tracking and data relay to a destination. The number of rounds describes the overall sensor’s schedule. The total number of charge requests efficiently fulfilled by the network is defined in the first series of studies to assess the charge throughput of CETS, ZCCS, NaZCCS, MPSA, and DMCP at various nodes densities. Figure 10 corresponds to charging throughput for CETS at different node densities, Figure 11 provides a comparative analysis of energy overhead for CETS at various node densities whereas from Figure 12, Figure 13 and Figure 14 corresponds to charging throughput for CETS at different node densities. Based on these analyses, it is determined that the difference in performance between CETS and the ZCCS, NaZCCS, MPSA, and DMCP is rarer when the density of the node is low. Both systems have comparable charging throughput. This difference, however, grows as node densities increase. The results show that CETS outperforms ZCCS, NaZCCS, MPSA, and DMCP in aspects of charging throughput at high densities because it uses on-demand subnode charging, and it can start serving a greater hop count per unit time. Figure 12 compares all the schemes with node density of 0.1. Figure 13 compares all the schemes with node density of 0.2. Figure 14 compares all the schemes with node density of 0.3. Figure 13 and Figure 14 also show that a change in node density has no significant effect on the charging throughput of NaZCCS; whereas in the case of ZCCS, MPSA, and DMCP, its outcomes in a substantial improvement in charging throughput; however, in the scenario of CETS, it results in a more substantial improvement throughout charging throughput. Based on the number of rounds the charging throughputs are calculated at hop densities 0.1, 0.2 and 0.3 and represented in the Table 8, Table 9 and Table 10.

### 4.5. Energy Analysis

**Figure 15:**
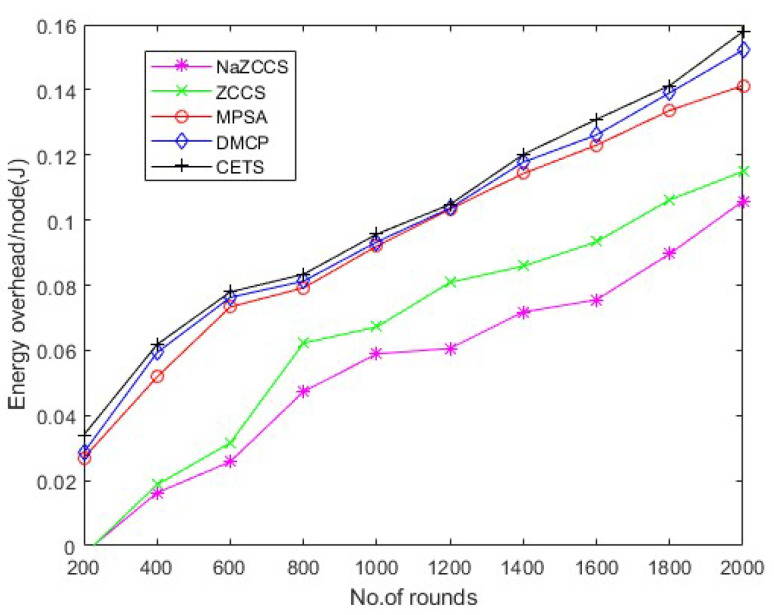
CETS versus other models on energy overhead per round at hop density 0.1.

**Figure 16:**
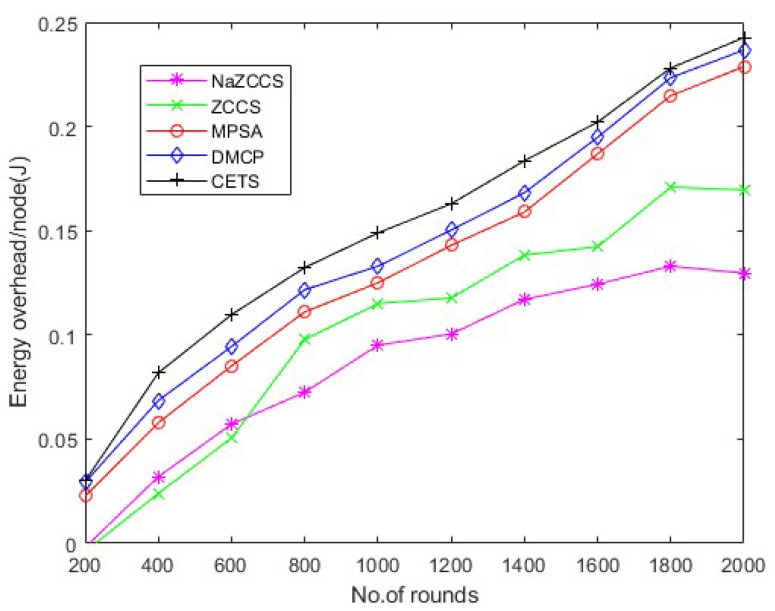
CETS versus other models on energy overhead per round at hop density 0.2.

**Figure 17:**
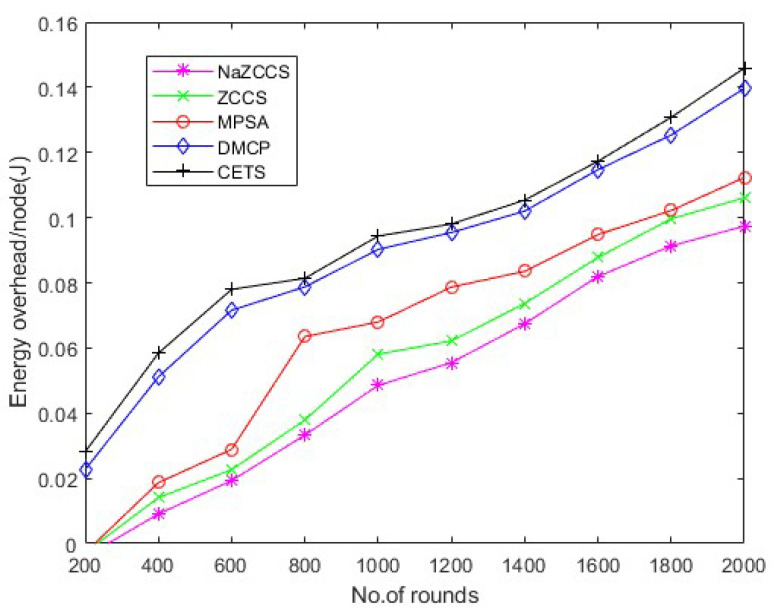
CETS versus other models on energy overhead per round at hop density 0.3.

Figure 15, Figure 16 and Figure 17 depict the energy overhead accumulated by all CETS, ZCCS, NaZCCS, MPSA, and DMCP under various densities of a node. For a node density of 0.1, Figure 15 compares the Energy overhead of all schemes. Figure 16 provides a comparative analysis of three methods with 0.2 node density. Figure 17 presents a comparative analysis with 0.3 node density, and all the methods have comparable overhead energy. However, as node density increases, the energy overhead accumulated by CETS exceeds that of ZCCS, NaZCCS, MPSA, and DMCP. This overhead is caused by control packets sent before beginning the recharging process. The Energy overhead based on various rounds at hop density 0.1 is shown in Table 11, at hop density 0.2 is shown in Table 12, and at hop density 0.3 is shown in Table 13.

### 4.6. Charging Latency Analysis

The charging effectiveness of an energy recharging mechanism is reflected in the average charging latency. A low charging response time indicates a high likelihood of connectivity and a more extended network lifetime. For all methods, average charge latency is analyzed using hops of 100 deployed over 60 × 60 areas divided into four 15 × 15 regions for five simulation runs. The average charging response time reported by the NaZCCS is double the average required to charge latency witnessed by the ZCCS, MPSA, and DMCP and even more so compared to CETS. CETS achieves this better charging latency improvement due to its on-demand subnode charging, which each region’s base station supports.

## 5. Conclusions

This research presents Wireless Powered-Internet of Everything (WP-IoE) outfitted with communication devices such as RF, Bluetooth, and Wi-Fi. IoE devices must have the necessary energy resources to extend their life span. This research proposes a two-phase energy transmission schedule to address the requirements. The proposed algorithm takes advantage of energy transfer to the needy node from the BS or neighbouring nodes to reduce the charge time of an IoE deployment while improving charge throughput. The cooperative energy transmission schedule uses the optimal path to receive the energy. Hence the additional UAVs or specific energy transmitter devices are not required to provide the energy. Experiment results show that CETS outperforms other models regarding charging throughput and latency. The clustering approach will be used to overcome data aggregation issues and charge overhead in future work.

## Figures and Tables

**Table 1:** The Base Station node BS’s initial tree routing table.

Current Hop	Neighbour Hop	No. of Communication Modules	Status	Hop Positioned	Traversed From
BS	N1	3	Unvisited	NA	NA
BS	N2	1	Unvisited	NA	NA
BS	N7	2	Unvisited	NA	NA

**Table 2:** The updated tree routing table of BS.

Current Hop	Neighbour Hop	No. of Communication Modules	Status	Hop Positioned	Traversed From
BS	N1	3	Visited	Right	BS
BS	N2	1	UnVisited	NA	BS
BS	N7	2	visited	Left	NA

**Table 3:** The updated left subTree routing table of N2.

Current Hop	Neighbour Hop	No. of Communication Modules	Status	Hop Positioned	Traversed From
N7	BS	3	Visited	Parent Hop	Root
N7	N2	1	Unvisited	NA	NA
N7	N8	2	visited	Left	N7
N7	N10	2	visited	Right	N7

**Table 4:** The updated right subtree routing of N2.

Current Hop	Neighbour Hop	No. of Communication Modules	Status	Hop Positioned	Traversed From
N1	BS	3	Visited	Parent Hop	Root
N1	N2	1	Unvisited	NA	NA
N1	N3	2	visited	Left	N1
N1	N5	1	UnVisited	NA	NA
N1	N8	2	Visited	Right	N1

**Table 5:** Characteristics of CPS.

Criterion	End Node	Intermediate Relay Nodes	Access Point
Energy Transmission	$E_{T}^{EN}$	$E_{T}^{RN}$	$E_{T}^{AP}$
Energy Reception	$E_{R}^{EN}$	$E_{R}^{RN}$	$E_{R}^{AP}$
Idle Power	$P_{I}^{EN}$	$P_{I}^{RN}$	
Transfer Power	$P_{T}^{EN}$	$P_{T}^{RN}$	
Computational Power	$P_{com}^{EN}$	$P_{com}^{RN}$	$P_{com}^{AP}$
Transmission Time	$T_{t}^{EN}$	$T_{t}^{RN}$	$T_{t}^{AP}$
Reception Time	$T_{r}^{EN}$	$T_{r}^{RN}$	$T_{r}^{AP}$
Idle Time	$T_{i}^{EN}$	$T_{i}^{RN}$	$T_{i}^{AP}$
Computational Time	$T_{c}^{EN}$	$T_{c}^{RN}$	$T_{c}^{AP}$

**Table 6:** Neighbours energy availability at node N5.

Current Hop	Neighbour Hop	Neighbour Hop Energy	No. of Communication Modules	Energy Transferring Hop
N5	N2	40%	2	NA
N5	N9	70%	3	N9

**Table 7:** Operating Voltage, Transmission, Receiving, and Data Rates of Various Devices.

Node	Transmission Rate	Receiving Rate	Data Rate	Operating Voltage
WDM	500 mA	380 mA	2 to 4 Mbps	3.0 to 3.6 V
RF (Rfbee v1.1)	35.4 mA	18.1 mA	4800 bps to 76,800 bps	3.0 to 3.6 V
BLUETOOTH (BM70/71)	3.3 mA	3.2 mA	8.6 kbps	1.9 to 3.6 V
Wi-Fi (ESP8266)	802.11b: +20 dBm 802.11 g: +17 dBm 802.11n: +14 dBm	802.11b: −91 dBm (11 Mbps) 802.11g: −75 dBm (MCS7)	2 to 4 Mbps	3.0 to 3.6 V

**Table 8:** CETS versus other models on charging throughput per round at hop density 0.1.

No of Rounds	NaZCCs	ZCCS	MPSA	DMCP	CETS
200	2.184585751	104.0614503	461.1824951	537.5725259	677.7383665
400	155.5988819	334.1711494	870.592657	882.9249959	1061.191891
600	321.909281	641.0232318	1522.397876	1509.149421	1572.384957
800	705.550727	986.3522116	2059.242207	2058.514012	2147.588734
1000	1101.994315	1471.917503	2698.315755	2582.5562	2671.560452
1200	1498.367433	1868.408071	3119.893824	3272.767847	3387.094501
1400	2265.438914	2546.146438	3503.629231	3733.034225	3808.977943
1600	2930.234197	3376.946748	3874.609476	4155.011628	4205.374551
1800	3339.315496	3952.361937	4168.964788	4487.773367	4563.576143
2000	4106.903761	4387.869677	4783.162247	4795.283174	4884.099504

**Table 9:** CETS versus other models on charging throughput per round at hop density 0.2.

No of Rounds	NaZCCs	ZCCS	MPSA	DMCP	CETS
200	1156.610389	1800.063543	2529.132085	3258.557358	4202.467016
400	2043.353938	3165.628989	3636.781174	3852.692481	4755.132185
600	3146.008796	3629.646558	4439.871354	4532.442993	5433.812505
800	4847.257911	5242.248073	5331.430769	4868.126662	6196.146193
1000	5139.687972	6476.447407	6565.451738	5762.004827	6873.221223
1200	5689.67766	7410.190238	7925.487857	6823.724827	8106.528731
1400	5936.713729	8343.933068	8817.404003	7845.045065	9424.916531
1600	6785.911364	9064.975168	9452.652349	8823.468427	10,443.38292
1800	6317.256294	9616.035049	10002.64204	9798.502845	11,248.25676
2000	6433.639713	10,165.48964	10,680.43053	11,152.83127	12,095.49237

**Table 10:** CETS versus other models on charging throughput per round at hop density 0.3.

No of Rounds	NaZCCs	ZCCS	MPSA	DMCP	CETS
200	−333.516605	−327.034561	141.0616276	−231.96458	658.3904853
400	542.0223592	383.3666117	1901.708289	1322.645677	1496.426194
600	1164.452933	1176.491015	3030.972982	2664.428818	2925.253928
800	2003.105979	2469.658825	4328.462154	4809.8311	4521.071466
1000	2753.171089	3681.02941	5584.435377	6786.082897	6330.02479
1200	3463.572262	4764.76546	6845.347301	8213.675956	7971.370972
1400	4002.044931	5306.016147	7133.489597	9853.478793	9522.894541
1600	4584.502899	5457.263844	8180.648397	11,702.71339	11,287.86257
1800	4990.40233	6672.647124	9612.562819	13,043.57053	12,925.50472
2000	6242.054191	6789.478253	11,545.44665	14,259.26247	14,726.43266

**Table 11:** CETS versus other models on energy overhead per round at hop density 0.1.

No of Rounds	NaZCCs	ZCCS	MPSA	DMCP	CETS
200	−0.00239116	−0.0028106	0.026943167	0.028613443	0.033965544
400	0.016234455	0.01870454	0.05178834	0.059272261	0.061769548
600	0.025778784	0.031564616	0.073333553	0.076258416	0.077919812
800	0.047308963	0.06219562	0.079182353	0.0812497	0.083352171
1000	0.058898666	0.067183899	0.091969241	0.093278397	0.095682332
1200	0.060573453	0.080849796	0.103352371	0.103658616	0.104806461
1400	0.071752728	0.085859874	0.114290687	0.11778049	0.120147897
1600	0.075505964	0.093354318	0.123006643	0.126144171	0.130929523
1800	0.089643929	0.106210636	0.133679306	0.13909517	0.141306736
2000	0.105837793	0.114935612	0.141316367	0.152221554	0.157918544

**Table 12:** CETS versus other models on energy overhead per round at hop density 0.2.

No of Rounds	NaZCCs	ZCCS	MPSA	DMCP	CETS
200	−0.00199468	−0.00398936	0.022606383	0.029255319	0.029920213
400	0.031914894	0.02393617	0.057845745	0.068484043	0.081781915
600	0.057180851	0.050531915	0.085106383	0.094414894	0.109707447
800	0.072473404	0.097739362	0.111037234	0.121675532	0.13231383
1000	0.095079787	0.115026596	0.125	0.132978723	0.14893617
1200	0.100398936	0.11768617	0.142952128	0.150265957	0.162898936
1400	0.117021277	0.138297872	0.158909574	0.168218085	0.183510638
1600	0.124335106	0.142287234	0.186835106	0.19481383	0.20212766
1800	0.132978723	0.17087766	0.214760638	0.223404255	0.228058511
2000	0.129654255	0.151595745	0.228723404	0.236702128	0.24268617

**Table 13:** CETS versus other models on energy overhead per round at hop density 0.3.

No of Rounds	NaZCCs	ZCCS	MPSA	DMCP	CETS
200	−0.0041304	−0.002854	−0.0028212	0.022677191	0.028149075
400	0.009066001	0.014073177	0.018671786	0.051126223	0.058410785
600	0.01930402	0.022665002	0.02888893	0.071597808	0.077982256
800	0.033275801	0.037880375	0.063518667	0.078745969	0.081382267
1000	0.048529944	0.058093978	0.067971147	0.09025062	0.094355818
1200	0.055496449	0.062167714	0.078677378	0.095431586	0.098042944
1400	0.067425397	0.073655291	0.083541406	0.102007418	0.105394159
1600	0.081954856	0.08775829	0.094802334	0.114705784	0.117297348
1800	0.09133101	0.099627593	0.102174424	0.125346405	0.130777059
2000	0.097393898	0.106096065	0.112331923	0.139682019	0.145897001

## Data Availability

Not applicable.
